# Dual Antibiotic-Infused Liposomes to Control Methicillin-Resistant *Staphylococcus aureus*

**DOI:** 10.3390/medicines12020014

**Published:** 2025-05-22

**Authors:** Sourav Chakraborty, Piyush Baindara, Surojit Das, Suresh K. Mondal, Pralay Sharma, Austin Jose T, Kumaravel V, Raja Manoharan, Santi M. Mandal

**Affiliations:** 1 Department of Bioscience and Biotechnology, Indian Institute of Technology Kharagpur, Kharagpur 721302, India; souravchakraborty68323@gmail.com (S.C.);; 2 Animal Science Research Center, Division of Animal Sciences, University of Missouri, Columbia, MO 65211, USA; piyush.baindara@gmail.com; 3 Department of Chemistry, Indian Institute of Technology Kharagpur, Kharagpur 721302, India; surojitdas2011@gmail.com; 4 National Institute of Homoeopathy, Block-GE, Sector-III, Salt Lake, Kolkata 700106, Indiaaustinjose.nih@gov.in (A.J.T.); kumaravel.nih@gov.in (K.V.); rajamanoharan.nih@gov.in (R.M.)

**Keywords:** antibiotics, resistance, liposome, MRSA, ampicillin, amikacin, tocopherol

## Abstract

**Background**: Methicillin-resistant *Staphylococcus aureus* (MRSA) considered under the category of serious threats by the Centers for Disease Control and Prevention (CDC), urges for new antibiotics or alternate strategies to control MRSA. **Methods**: Ethosome-like liposomes have been developed and characterized using dynamic light scattering (DLS), Fourier transform infrared spectroscopy (FTIR), and scanning electron microscopy (SEM). Liposomes were confirmed for antibiotics infusion by encapsulation efficiency and release kinetics as well. Further, the antimicrobial potential of liposomes was checked by determination of minimum inhibitory concentrations (MICs), crystal violet assay, and live/dead biofilm eradication assay. **Results**: The specially designed liposomes consist of amphiphilic molecules, tocopherol, conjugated with ampicillin and, another antibiotic amikacin, loaded in the core. The developed liposomes exhibited good encapsulation efficiency, and sustained release while serving as ideal antibiotic carriers for advanced efficacy along with anti-inflammatory benefits from tocopherol. Conclusively, newly designed liposomes displayed potential antimicrobial activity against MRSA and its complex biofilms. **Conclusions**: Overall, dual antibiotic-encapsulated liposomes demonstrate the potential to eradicate MRSA and its mature biofilms by dual-targeted action. This could be developed as an efficient anti-infective agent and delivery vehicle for conventional antibiotics to combat MRSA.

## 1. Introduction

In the recent past, the rapid emergence of drug resistance became a serious concern worldwide causing millions of deaths worldwide each year [1,2]. MRSA is one of the most serious concerns in healthcare throughout the world as it is resistant to several conventional antibiotics resulting in longer hospital stays and higher death rates [3]. The major causes of antibiotic resistance are as follows: overuse and misuse of antibiotics; incomplete treatment; excessive use in livestock and crops; poor infection control strategies in healthcare settings; horizontal gene transfers in bacterial strains; and lack of new antibiotics [4]. Addressing antibiotic resistance requires a multifaceted approach that involves better stewardship of existing conventional antibiotics, sustained delivery, developing new antibiotics, and efficient strategies using existing resources [5]. Several approaches, ranging from antimicrobial peptides (AMPs) to antimicrobial nanoparticles have been demonstrated to battle against the rapid emergence of drug resistance; however, in the current scenario, an efficient delivery system is very crucial for sustained release and quick penetration to the targeted bacteria or biofilms [6,7,8]. Liposomes have risen as one of the most promising strategies in the recent past for the targeted delivery of antimicrobial agents to combat resistance [9,10]. Notably, liposomes have been demonstrated as an efficient delivery system for a different class of antimicrobial agents including, proteins, nucleotides, plasmids, and conventional antibiotics [11,12]. Interestingly, recent technical advancements in liposome synthesis make them able to undergo various clinical trials, suggesting the drug-delivery potential and applications of liposomes in future therapeutics [13,14]. Importantly, liposomes can form lipid bilayer-like structures and can easily fuse with target cell membranes for efficient drug delivery. Further, over time, liposomes can improve the efficiency of antimicrobial drugs by shielding them from lipid bilayer breakdown, enzymatic degradation, and chemical degradation via adhesion and fusion [10,15]. By considering the various advantages of the liposomal drug delivery system, here we have developed specially designed liposomes containing ampicillin conjugated with tocopherol and loaded with amikacin as a second antibiotic. Tocopherol is conjugated with ampicillin because of its potential anti-inflammatory properties, while dual antibiotics are loaded for efficient eradication of MRSA and its mature biofilms. The developed dual-antibiotic encapsulated liposomes demonstrate efficient eradication of MRSA and its biofilms; however, further in vivo and toxicity studies using appropriate animal models are warranted.

## 2. Material and Methods

### 2.1. Liposome Preparation

**Figure 1:**
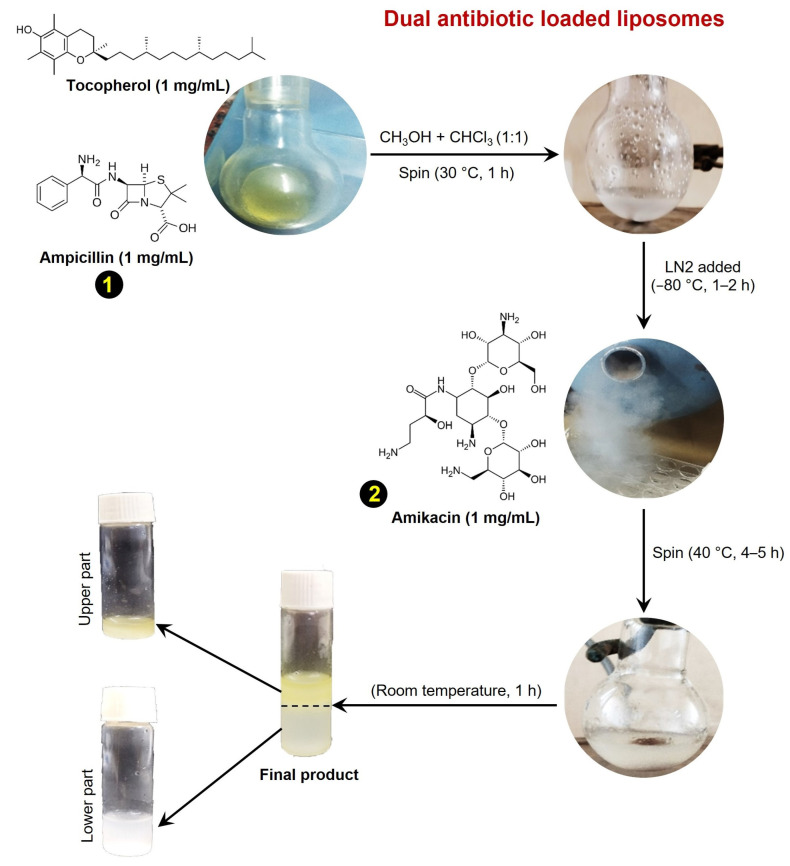
Schematic diagram of the synthesis procedure of dual antibiotic-loaded liposomes. LN2 denotes liquid nitrogen. Yellow-colored numbers 1 and 2 in black circles represent the sequential process for the addition of ampicillin and amikacin, respectively.

To check the efficacy of encapsulated conventional antibiotics against drug-resistant bacteria, we attempted the preparation of antibiotic-containing liposomes as described earlier [11]. We chose β-lactam antibiotic ampicillin in combination with tocopherol (vitamin E) for the liposome preparation. A mixture of 1 mg/mL ampicillin in 5 mL methanol (Sigma, Ronkonkoma, NY, USA) and 1 mg/mL of tocopherol in 5 mL chloroform (Sigma, USA) was rotated in a round-bottom flask for 2 h at 40 °C. Subsequently, liquid nitrogen was added to the mixture 3 to 4 times to remove any moisture content and immediately refrigerated at −84 °C overnight. Next, 1 mg/mL of amikacin dissolved in 70% ethanol was added to the mixture and rotated for 3–4 h at 40 °C. Finally, rotary dialysis was used to prepare the liposomes [16]. The mixture solution was rotated for 6 h and subsequently dialyzed for 24 h against distilled water using a dialysis cassette (7 kDa, MWCO, Thermos Scientific, Waltham MA, USA), and subsequently lyophilized and stored at 4 °C (Figure 1). Ampicillin, amikacin, and tocopherol were purchased from Sigma, USA with at least >98% purity.

### 2.2. DLS

DLS measurements of prepared liposomes were performed using Malvern Zetasizer Nano ZS devices (Malvern Devices Ltd., Worcester, UK) equipped with a titration device MPT-2 (Malvern Instruments Ltd., Worcester, UK) and a 4 mV He-Ne laser illuminating at 633 nm [17]. To guarantee laser stability, the whole setup was turned on at least half an hour before the measurements were performed. The quartz cells were pre-treated with analytical-grade ethanol and thoroughly washed with pure water for equilibration. Next, the quartz cells were equilibrated with samples before the final loading and measurements to avoid dilution with reaming water content. Liposome samples were dissolved and suspended in PBS (Fisher Scientific, Waltham, MA, USA) for analysis and analyzed for size using light scattering at 25 °C. Three independent measurements were made for each set of liposome preparations, and the mean value of the triplicates was considered as the final result.

### 2.3. Encapsulation Efficacy

After size measurements of antibiotic-loaded liposomes, encapsulation efficiency was determined, as described earlier, with minor modification [18]. Encapsulation efficiency was calculated as the percentage difference between the total antibiotics (encapsulated and non-encapsulated) and the free antibiotics (non-encapsulated). Two distinct methods were used to measure the total and free amount of antibiotics. First, to estimate the total amount of antibiotics, 0.5% methanol was added to the liposome mixture and incubated for 50 min at 4 °C. Next, five parts of PBS were added to the suspension mixture and analyzed using a UV-Vis spectrophotometer (Thermo Scientific, Waltham, MA, USA). The total amount of antibiotics was calculated by measuring the absorbance at 210 nm and 240 nm for amikacin and ampicillin, respectively. Second, the free antibiotic amount was determined by centrifuging the liposomes at 20,000 g for 30 min. Subsequently, released antibiotics in supernatant were measured by UV-Vis spectrophotometer as described above. Finally, following the measurement of the total and free amount of antibiotics, the percentage of encapsulation efficiency was determined as follows, encapsulation efficiency (%) = (Total drug − Free drug) × 100/Total drug.

### 2.4. Antibiotics Release Kinetics

For the efficient delivery of antibiotics via liposomes, an effective release is a must. To confirm the release efficacy of encapsulated antibiotics (ampicillin and amikacin) from liposomes, a dialysis approach was employed [19]. PBS of pH 5.0 and pH 8.0 was used for dialysis to check the release efficiency of antibiotics, while regenerated cellulose dialysis cassettes with 30 mm × 25 mm release areas (12–14 kDa, MWCO, Thermo Scientific, USA) pre-soaked in different pH buffer solutions were used. Then, 1 mL of liposomes formulated solution containing encapsulated antibiotics was added to dialysis tubing in respective pH buffer conditions with 150 rpm rotation at 37 °C. Respective sample volumes were simultaneously refilled with fresh buffer after each 1 mL sample was obtained at various intervals. Antibiotic concentrations were determined in the solution at 210 nm and 240 nm for amikacin and ampicillin, respectively, by employing a UV-Vis spectrophotometer.

### 2.5. FTIR

A NexusTM 870 FTIR spectrophotometer fitted with a deuterated triglycine sulfate detector thermoelectric cool (DTGS TEC) (Thermo Nicolet, Waltham, MA, USA) was used to record infrared spectra. A CaF_2_ cell was used to capture the solution spectra of three identical samples. The water combination mode was detected at a flat baseline around 2200 cm^−1^.

### 2.6. SEM

SEM was performed to visualize and estimate the size of antibiotic-encapsulated liposomes. By using the drop-cast method approximately 5 to 10 μL of liposome solution in PBS was directly spotted on the glass coverslip to capture the FE-SEM images. Additionally, a low vacuum was maintained in an auto sputter coater (E5200, Bio-Rad, Hercules, CA, USA) gold for up to 120 s, while the samples were dried and glued to a graphite stub. Samples were finally scanned, visualized, and examined for surface morphology by using AFM and SEM (JEOL JSM 5800, Peabody, MD, USA) operating at an accelerated voltage of 5–20 kV. The bacterial samples for SEM were prepared using 10 µL of treated MRSA with antibiotic-encapsulated liposomes. The samples were directly placed on a copper grid and allowed to dry for 10 min, and subsequently fixed with modified Karnovskys fixative for 2 h. Next, samples were further fixed with 2% OsO_4_ (Sigma Aldrich, St. Louis, MO, USA) for 10 min in a closed chamber and then gradually dehydrated in graded ethanol (30–100%), following freeze drying. Finally, overnight freeze-dried samples were directly mounted on aluminum stubs for imaging [20]. The treated MRSA samples along with controls were visualized and imaged under a ZEISS EVO 60 Scanning Electron Microscope, equipped with an Oxford EDS Detector (Zeiss, Hebron, KY, USA).

### 2.7. Bacterial Strain and Antibacterial Assay

A clinically isolated methicillin-resistant strain of *Staphylococcus aureus* (MRSA) was obtained from the Department of Microbiology, Bankura Sammilani Medical College and Hospital, Kenduadihi, Bankura722102, West Bengal, India. The MRSA was grown on nutrient agar (NA) and nutrient broth (NB), for maintaining the single colonies and liquid cultures, respectively. Next, antibiotics-encapsulated liposomes were tested for antimicrobial efficacy against MRSA by employing a well-diffusion assay [21]. First of all, MRSA was grown on NA plates overnight to obtain the single colonies. A single colony was then inoculated in 5 mL NB for 2–4 h and allowed to grow until 0.2–0.4 OD, which was then used to prepare bacterial lawn. Wells were punched on the NA plates with the help of a core borer (HiMedia, Thane, India) and subsequently loaded with 100 μL of test dilutions. Plates were then incubated overnight at 37 °C. Antibiotics alone and PBS were used as controls. A zone of inhibition encircling the bored well was observed when the growth of MRSA was inhibited by test dilutions. All bacterial mediums and antibiotics were purchased from Hi Media, India, and Sigma, USA, respectively.

### 2.8. MIC

The broth microdilution assay was used to determine the MICs of ampicillin and liposomes against MRSA with some modifications as described earlier [22]. The individual drugs, ampicillin, amikacin, tocopherol, and antibiotic-loaded liposome were used in concentrations ranging from 1 to 512 µg/mL to explore the antimicrobial potential. PBS was used to prepare the serial dilution of antibiotics and antibiotic-encapsulated liposomes. An assay was performed using 96 well Elisa plates and the results were recorded after 18 h of incubation at 37 °C.

### 2.9. Biofilm Eradication

The biofilm eradication potential of antibiotic-encapsulated liposomes was tested as described earlier with modifications [23]. Statically formed biofilms were allowed to mature for 48–60 h at 37 °C and subsequently washed with PBS to remove the non-adherent cells. The biofilms were then treated with respective test dilutions of antibiotic-encapsulated liposomes and further incubated overnight at 37 °C. Antibiotics and PBS alone were used as controls. After incubation, the amount of persisted biofilm was measured using 0.1% crystal violet (CV) solution (Sigma-Aldrich, St. Louis, MO, USA) and following resuspension in 70% ethanol with gentle mixing. Finally, absorbance was recorded at 595 nm to determine the antibiofilm potential of liposomes.

### 2.10. Leakage Assay

DNA leakage assay was used to determine the antimicrobial potential antibiotic encapsulated liposomes against MRSA as described earlier [24]. Different time intervals of 0, 5, 10, 20, and 30 min were used to monitor the DNA release upon treatment with different dilutions of liposomes and subsequently, the OD was recorded at 260 nm using a spectrophotometer [23].

### 2.11. Statistical Analysis

All the experiments in the present study were performed three times independently in triplicates, including the controls wherever applicable. The results and data presented are based on the mean ± standard deviation of the mean (SD). Column statistics for non-parametric data were analyzed using a one-sample *t*-test, wherever applicable. All the results for all the corresponding experiments were finalized and considered significant only when *p* < 0.05 in all independent experiments.

## 3. Results and Discussion

### 3.1. Synthesis of Dual Antibiotic-Loaded Tocopherol Conjugated Liposomes

Dual antibiotic-encapsulated tocopherol-conjugated liposomes were synthesized in a sequential process (Figure 1 and Figure S1). Alpha-tocopherol is a lipid-soluble vitamin and can change the fluidity of bacterial membranes making them susceptible to antibiotics [25]. Next, ampicillin is a β-lactam antibiotic that belongs to the penicillin family and restricts bacterial growth by inhibiting cell wall synthesis. Resistance against ampicillin is well known and governed by β-lactamases production, alterations in penicillin-binding proteins, and efflux pumps that lower the intracellular drug concentrations and subsequently develop resistance [26]. On the other hand, amikacin is an aminoglycoside antibiotic in nature and a semisynthetic derivative of kanamycin. Amikacin functions by blocking the 30S ribosomal subunit of bacteria. However, a gene mutation that reduced the binding affinity of amikacin to bacterial ribosomes, is known to develop resistance against amikacin [27]. To develop an alternative approach for the efficient delivery of conventional antibiotics, we have followed a sequential synthesis approach to prepare the antibiotic-loaded liposomes as described above (Figure 1). Notably, ampicillin conjugated with tocopherol has both a polar head and a hydrophobic tail, which is formed when an amphiphilic conjugated structure is hydrated in an aqueous solution. Further, the hydrophobic tails of the lipids face inward and are shielded from water, while the polar heads face outward, and interact with the aqueous environment. This self-assembly process leads to the formation of lipid bilayers, which can then be enclosed to form a liposome-like structure. Eventually, the second drug, amikacin is solubilized in aqueous alcohol and enclosed in a water-soluble core of the liposome vesicles when rotated at 40 °C. Also, similar to the ethosomes, these liposomes contain high concentrations of ethanol that make them more flexible and deformable than traditional liposomes, making them better and more efficient drug-delivery agents [28,29].

### 3.2. Spectroscopic Analysis

**Figure 2:**
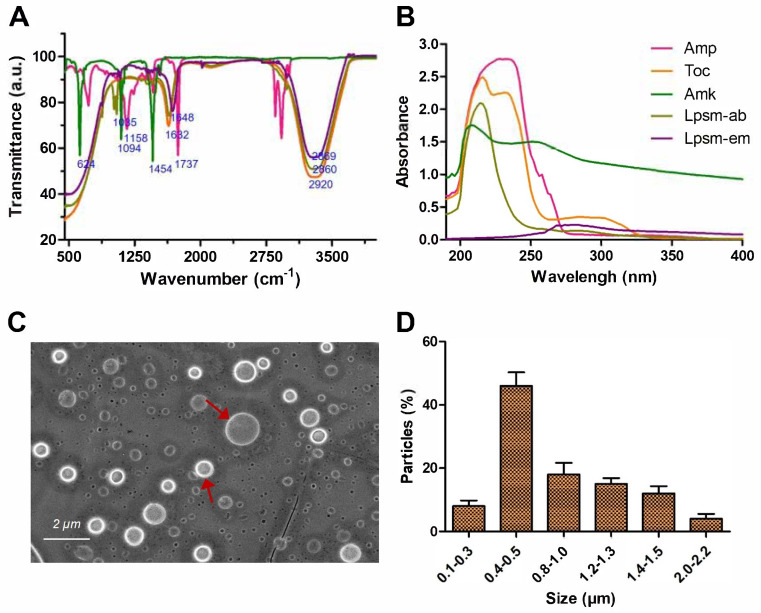
Characterization of antibiotics-encapsulated liposomes. (**A**) Overlaid view of FTIR spectroscopic spectra, and (**B**) UV-Vis absorbance spectra where ampicillin, tocopherol, amikacin, liposomes alone, and antibiotics-loaded liposomes are represented as pink, orange, green, purple, and light green color lines, respectively, while separately confirming their complex formation. Ampicillin, amikacin, tocopherol, liposomes alone, and liposomes loaded with antibiotics are denoted as Amp, Amk, Toc, Lpsm-em, and Lpsm-ab, respectively. (**C**) SEM images reveal the circular smooth structure of liposomes. Red solid arrows indicate the liposomes containing smooth round surfaces. (**D**) DLS analysis shows the distribution of particle sizes. Error bars represent standard deviation (SD), while the statistical significance is considered at the level of *p* < 0.05. All the experiments were performed three times independently in triplicates.

To confirm the conjugation of tocopherol and ampicillin, FTIR and UV-Vis spectrophotometry analysis is performed. Among the FTIR spectra obtained from ampicillin, tocopherol, and antibiotic-encapsulated liposome, a distinct difference was observed at peaks 1007 cm^−1^, 1065 cm^−1^, and 1632 cm^−1^. The corresponding peak for liposomes at 1007 cm^−1^ likely resembles the C–O stretching in the ether group (–O–) while the peak at 1065 cm^−1^ corresponds to the C–N stretching in the amide group (–CONH–) of ampicillin [30]. Additionally, the peak at 1737 cm^−1^ is attributed to the carbonyl (C=O) stretching in the β-lactam ring and amide group (–CONH–) of ampicillin. Characteristic peaks attributed to amikacin were observed at 624, 1094, and 1454 cm^−1^, corresponding to the N–H stretching, C–O stretching, and aminoglycoside moiety. Further, the characteristic peaks for antibiotic-loaded liposomes were observed at 2860, 2920, and 1632 cm^−1^ which correspond to the alkyl and ester groups, respectively (Figure 2A) [31]. Notably, liposomes alone exhibit a characteristic spectrum of 1648, and 2869 cm^−1^ for C=O and CH2 stretching, respectively [32]. Overall, FTIR spectra of antibiotic-encapsulated liposomes confirmed the conjugation of ampicillin and tocopherol. Additionally, the UV-Vis spectral overlay analysis of individual components of tocopherol, ampicillin, and amikacin along with antibiotic-encapsulated liposomes also confirmed the formation and distinct properties of liposomes (Figure 2B).

### 3.3. Microscopic Size Analysis of Liposomes

The SEM image revealed the circular structure of liposomes with smooth surfaces (Figure 2C). The basic structure resembles with liposome as a spherical vesicle with an aqueous core surrounded by a lipid bilayer membrane. DLS size investigation revealed that the majority of liposomes are in the range of 400–500 nm while the remaining are from 0.5 to 1 µm (Figure 2D).

### 3.4. Encapsulation and Release Kinetics Liposomes

**Figure 3:**
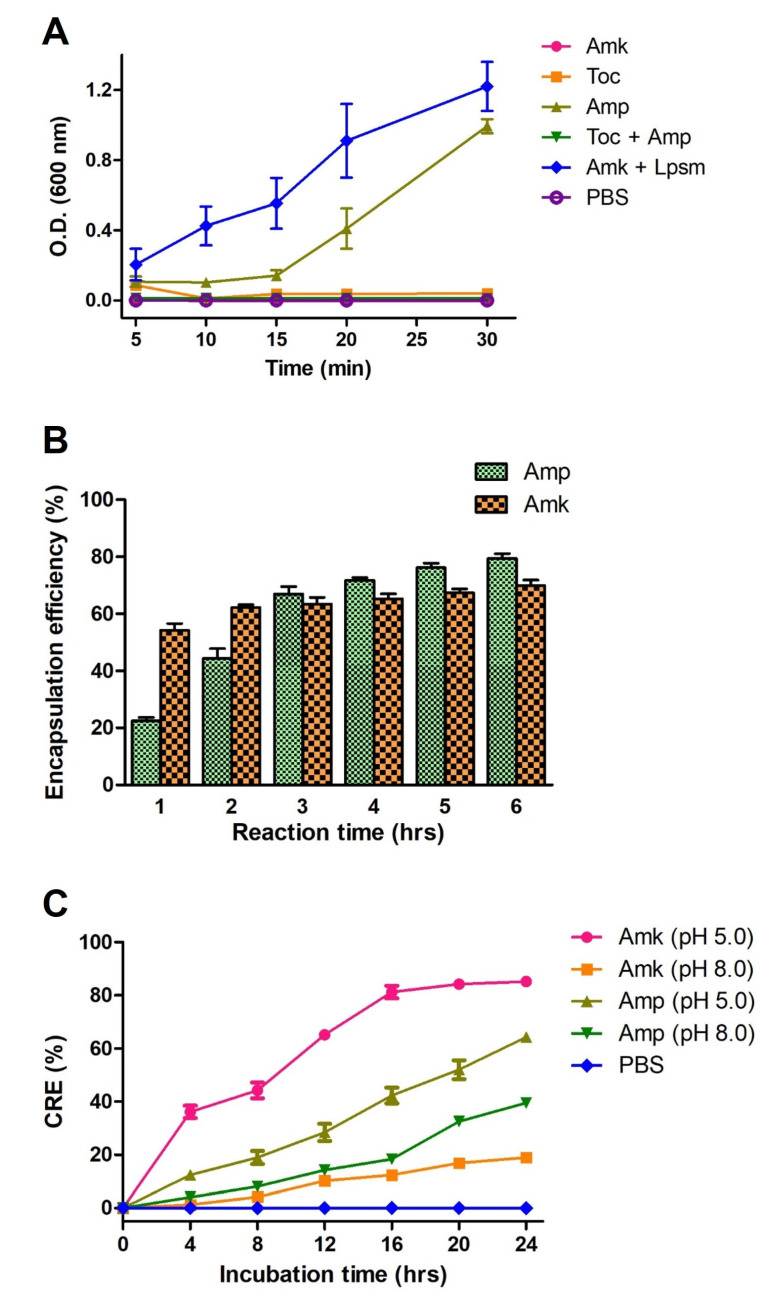
Determination of encapsulation and release efficiency of antibiotics encapsulated liposomes. (**A**) Turbidity measurement of ampicillin, amikacin, tocopherol, and tocopherol conjugated with ampicillin and amikacin combined with liposome (loaded with ampicillin and tocopherol) at an absorbance of 600 nm. (**B**) Rate and efficiency calculations of ampicillin and amikacin in liposomes at different reaction time points. (**C**) Release kinetics of liposome-encapsulated amikacin and ampicillin after acid and alkaline treatment at pH 5.0 and 8.0, respectively. PBS alone was used as a control. Ampicillin, amikacin, tocopherol, and liposomes are denoted as Amp, Amk, Toc, and Lpsm, respectively. Error bars represent SD while the statistical significance is considered at the level of *p* < 0.05. All the experiments were performed three times independently in triplicates.

The encapsulation and release efficiency of liposomes is governed by a complex interplay of factors including liposome formation methods, drug properties, liposome size, and composition. In the present study, liposomes are formulated in different ways where the membrane lipid is prepared by the conjugation of tocopherol and ampicillin after extensive rotation and phase change which is confirmed by the measurement of optical density (OD) at 600 nm for turbidity evaluation (Figure 3A). Notably, following the dialysis, encapsulation efficiency for both ampicillin and amikacin was determined in a time-dependent manner, and observed that a maximum of 70% encapsulation occurred at 3 h of stirring (Figure 3B). Further, both ampicillin and amikacin displayed release from liposomes upon acid and alkaline treatment; however, better release was observed at pH 5.0 (Figure 3C). This was probably due to the low pH affecting ester bonds between tocopherol and ampicillin through the promotion of protonation of the ester oxygen that enhances electrophilicity, and facilitates nucleophilic attack by water molecules, leading to hydrolysis of the ester bond. The liposome images were captured immediately to visualize the release in acidic conditions that also revealed the change in shape and loss of membrane integrity.

### 3.5. Antimicrobial and Antibiofilm Potential

**Figure 4:**
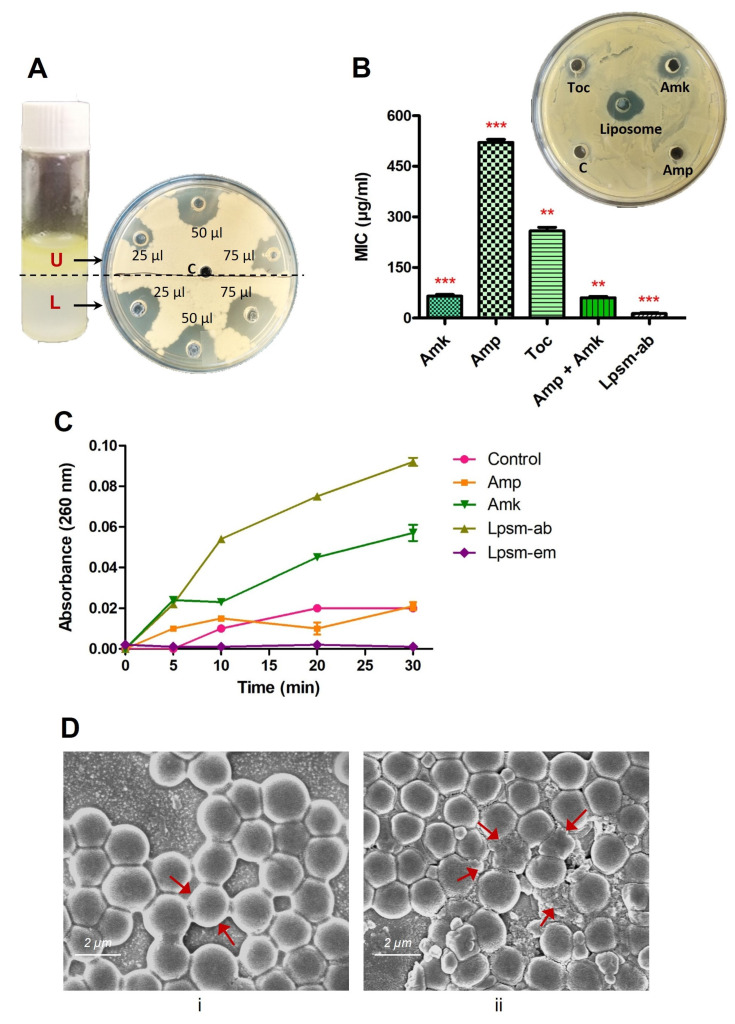
Antimicrobial potential of antibiotics encapsulated liposomes against MRSA. (**A**) Two layers (upper and lower, indicated in bold red font U and L) of final liposome preparation were tested separately with 25, 50, and 75 µL of volume using a well-diffusion assay to check efficiency against MRSA. The lower whitish part exhibits the highest and is used for further analysis. (**B**) MIC determination and comparison of antibiotics encapsulated liposomes with antibiotics alone. Well-diffusion assay image (inset) showing the corresponding results. (**C**) Intracellular leakage of MRSA upon treatment with antibiotics encapsulated liposomes and antibiotics. Treatment with PBS alone was used as a control. Ampicillin, amikacin, tocopherol, liposomes alone, and liposomes loaded with antibiotics are denoted as Amp, Amk, Toc, Lpsm-em, and Lpsm-ab, respectively. (**D**) SEM images of MRSA, untreated (**i**) and treated (**ii**) with antibiotic-encapsulated liposomes at a concentration of five µg/mL for one h. Intact cell membranes in untreated samples and membrane rupture in treated samples are indicated with solid red arrows. Error bars represent SD, while the statistical significance is considered at the level of *p* < 0.05. Two or three red stars indicate the statistical significance for the *p* values < 0.01 and 0.001, respectively. All the experiments were performed three times independently in triplicates.

**Figure 5:**
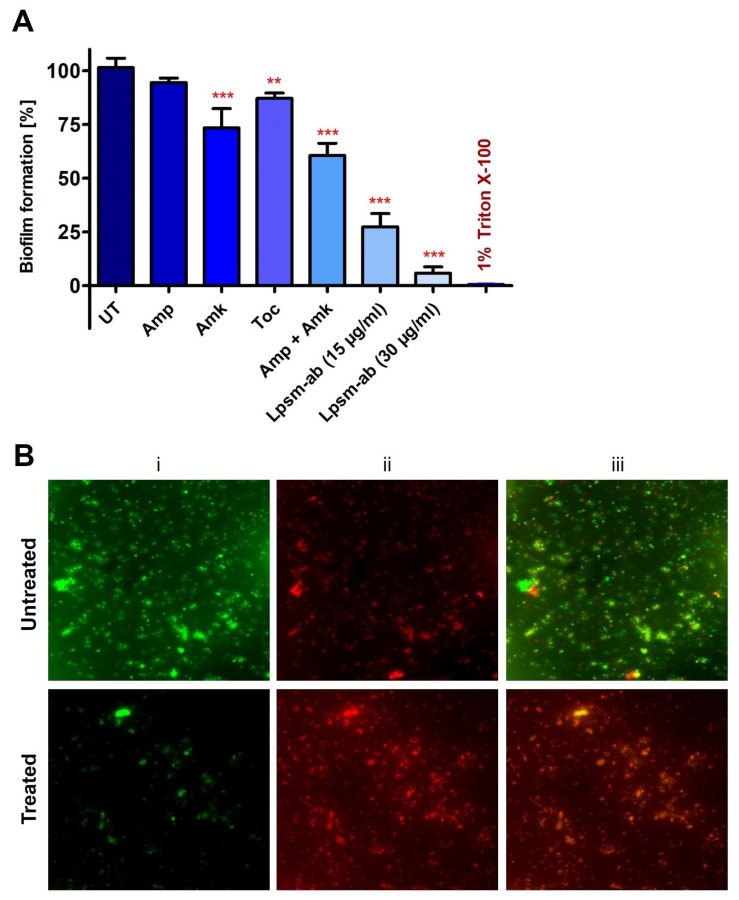
Biofilm eradication potential of antibiotics encapsulated liposomes against MRSA. (**A**) Crystal violet assay showing efficient eradication of MRSA biofilms in comparison to the individual antibiotics. PBS alone and 1% Triton X-100 treatments were used as negative and positive controls, respectively. Ampicillin, amikacin, tocopherol, liposomes alone, and liposomes loaded with antibiotics are denoted as Amp, Amk, Toc, and Lpsm-ab, respectively. (**B**) Fluorescence microscopic image of live/dead biofilm eradication assay of untreated biofilms (upper panel), and treated biofilms (lower panel) for 1 h at 15 µg/mL of antibiotics encapsulated liposomes (**i**) sytox green (**ii**) propidium iodide, and (**iii**) merged. Error bars represent SD while the statistical significance is considered at the level of *p* < 0.05. Two or three red stars indicate the statistical significance for the *p* values < 0.01 and 0.001, respectively. All the experiments were performed three times independently in triplicates.

The antibacterial activity of synthesized antibiotic-encapsulated liposomes was evaluated against MRSA. Interestingly, during the synthesis of liposomes, two phases with distinct physical appearances were visualized and subsequently separated to determine antimicrobial activity against MRSA. The upper yellowish phase and lower whitish phase of the synthesized liposome mixture were used for well diffusion assay in three different volumes of 25, 50, and 75 µL to check the antimicrobial potential (Figure 4A). Notably, the lower whitish phase exhibited efficient activity against MRSA in a concentration-dependent manner. Following the well-diffusion assay results, the lower whitish phase of the synthesized liposome mixture was used to determine the MIC values, using microtiter plate assay along with individual antibiotics. Interestingly, antibiotic-encapsulated liposomes exhibited potential antimicrobial activity against MRSA in comparison to the individual antibiotics and were able to completely restrict the MRSA growth at 15 µg/mL of concentration (Figure 4B). This suggests that liposomes provide an efficient way of antibiotic delivery in comparison to direct delivery of antibiotics. Further, SEM images confirmed the membrane-specific killing mechanism of antibiotic-encapsulated liposomes against MRSA within one hr of treatment with a sublethal dose of five µg/mL. SEM images revealed the membrane deformities in MRSA within 30 min of treatment with antibiotic-loaded liposomes. Cell membrane rupture, shrinkage in cell size, and clumping occurred upon liposome treatment (Figure 4D). Interestingly, tocopherol showed negligible activity while ampicillin exhibited no activity against MRSA. Notably, amikacin exhibits a MIC of 64 µg/mL against MRSA (Figure 4B). This suggests the potential of delivery efficacy for antibiotics-encapsulated liposomes against MRSA over individual antibiotics. Next, we performed a crystal violet assay followed by bacterial live/dead detection. Interestingly, antibiotic-encapsulated liposomes were efficiently able to deliver the antibiotics to biofilms and thus completely eradicated the mature biofilms of MRSA within 1 h of treatment (Figure 5A,B). Results confirmed that antibiotics-encapsulated liposomes efficiently eradicated about 90% of MRSA biofilms at 30 µg/mL of concentration.

Overall, the double antibiotic-encapsulated liposomes exhibited significant activity against MRSA and efficient eradication in comparison to the antibiotics alone. Additionally, tocopherol is known to have its own antibacterial and anti-inflammatory activities that might contribute to the efficacy of developed liposomes [33]. Tocopherol also facilitates liposomes to an efficient interaction and fusion with negatively charged bacterial membranes due to its amphiphilic nature and, thus, demonstrates the potential of dual mechanisms of action, including drug-delivery and antibacterial properties. Overall, antibiotic-encapsulated liposomes with tocopherol can passively permeate through the bacterial outer membrane and can directly deliver the drugs in periplasmic space for efficient eradication of drug-resistant bacteria. In conclusion, antibiotic-loaded and tocopherol-conjugated liposomes represent a significant advancement in engineered liposomal drug delivery to fight against the global challenge of drug-resistant microbial pathogens.

### 3.6. Conclusions and Future Directions

In the present study, we demonstrate the successful preparation of engineered liposomes containing dual antibiotics conjugated with tocopherol. Engineering antibiotic-encapsulated liposomes were also characterized by their size, encapsulation, and release efficiency. Importantly, these engineered antibiotic-loaded liposomes are promisingly able to eradicate MRSA and mature biofilms, when compared to individual antibiotics. Interestingly, our results were in corroboration with earlier studies of liposome-based delivery of antibiotics with improved efficacy [34,35]. Especially, the dual antibiotic encapsulation along with antibiofilm properties of tocopherol presents a relatively new approach to combating MRSA. Conclusively, newly synthesized and engineered liposomes represent a potential alternative for efficient drug delivery along with subsequent eradication of drug-resistant pathogens.

## Data Availability

The original contributions presented in this study are included in the article/Supplementary Materials, and further inquiries can be directed to the corresponding author/s.

## Supplementary Materials

The following supporting information can be downloaded at: https://www.mdpi.com/article/10.3390/medicines12020014/s1, Figure S1: (A) Tocopherol-ampicillin conjugate. (B) Tocopherol-ampicillin conjugate + amikacin complex.
