# Supplementary material for: Dual Antibiotic-Infused Liposomes to Control Methicillin-Resistant Staphylococcus aureus
Source: Medicines (Basel). 2025 May 22;12(2):14. doi: 10.3390/medicines12020014 (PMC12195063; doi:10.3390/medicines12020014)

Dual Antibiotic infused Liposomes to Control of methicillin-resistant *Staphylococcus aureus*

Sourav Chakraborty [1], Piyush Baindara [2],Surojit Das [3], Suresh K. Mondal [2], Pralay Sharma [4], Austin Jose T [4], Kumaravel V [4], Raja Manoharan[4], Santi M. Mandal [2,5]*

[1] Department of Bioscience and Biotechnology, Indian Institute of Technology Kharagpur, Kharagpur 721302, WB, India

[2] Animal Science Research Center, Division of Animal Sciences, University of Missouri, Columbia, MO 65211, USA

[3] Department of Chemistry, Indian Institute of Technology Kharagpur, Kharagpur 721302, WB, India

[4] National Institute of Homoeopathy, Block-GE, Sector-III, Salt Lake, Kolkata 700106, India

[5] Department of Chemistry and Biochemistry, University of California San Diego, 9500 Gilman Dr, La Jolla, CA 92093, USA

***Correspondence**

Santi M. Mandal, PhD

Department of Bioscience and Biotechnology,

Indian Institute of Technology Kharagpur

Kharagpur 721302, WB, India

Email: mandalsm@gmail.com

Figure S1. (A) Tocopherol-ampicillin conjugate + amikacin complex. (B) Tocopherol-ampicillin conjugate.

A

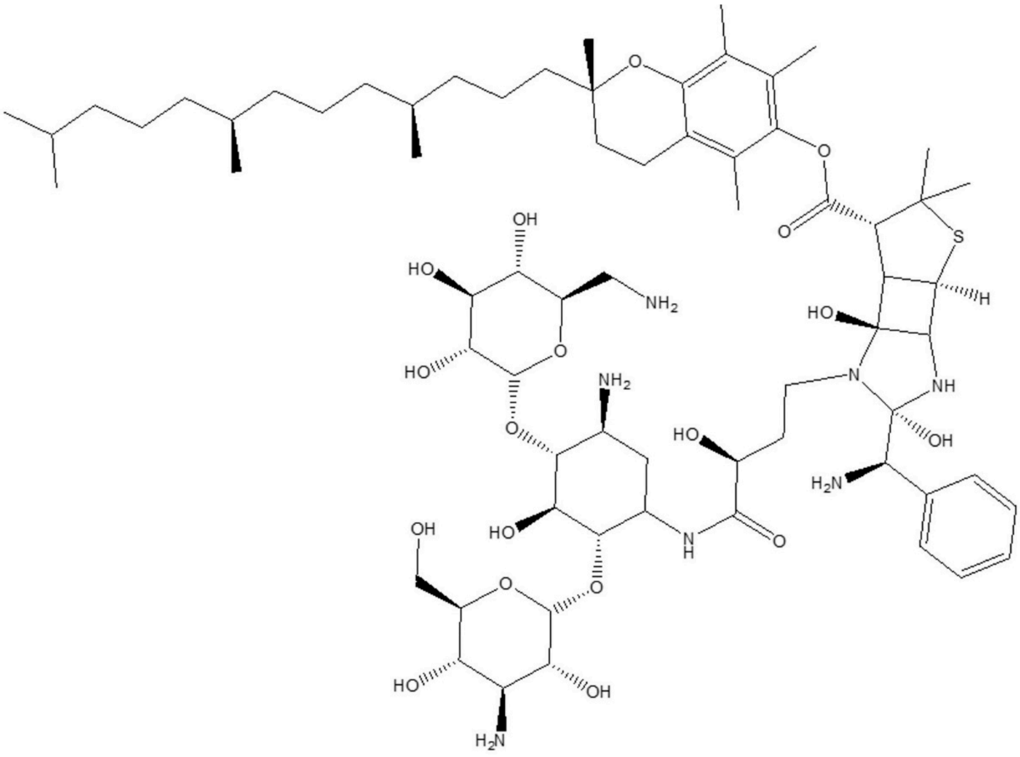

B

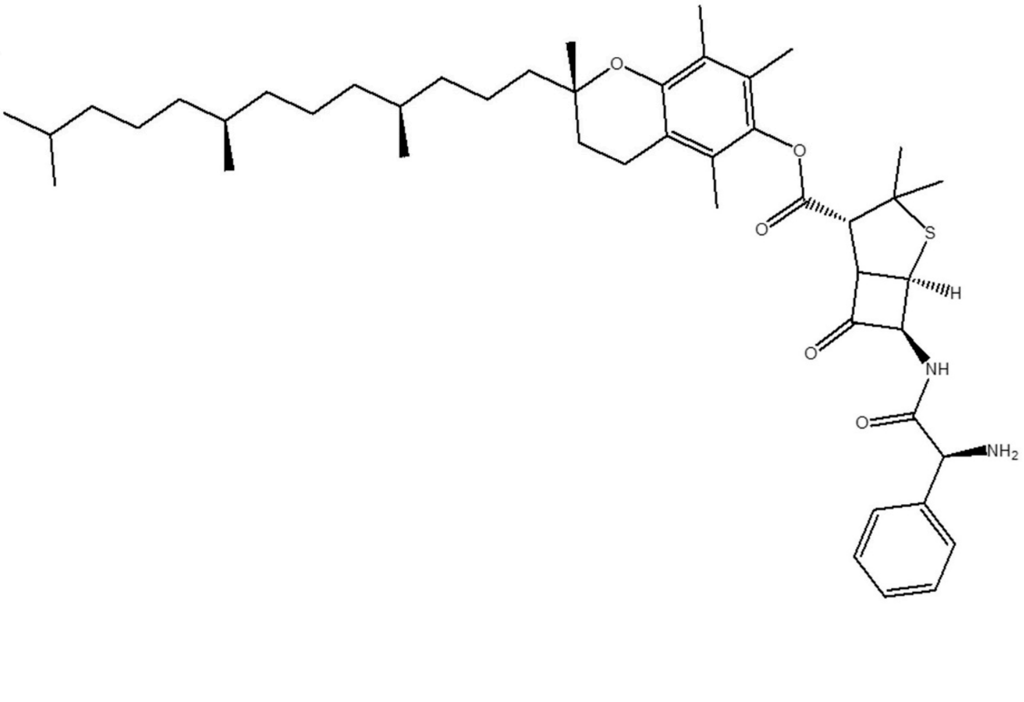

Supplement: Supplementary file 1 [file medicines-12-00014-s001.zip › medicines-3519554-supplementary.pdf]
